# Co-Occurrence of Cyanobacteria and Cyanotoxins with Other Environmental Health Hazards: Impacts and Implications

**DOI:** 10.3390/toxins12100629

**Published:** 2020-10-01

**Authors:** James S. Metcalf, Geoffrey A. Codd

**Affiliations:** 1 Brain Chemistry Labs, Jackson, WY 83001, USA; 2 School of Life Sciences, University of Dundee, Dundee DD1 5EH, UK; g.a.codd@stir.ac.uk; 3 Biological and Environmental Sciences, University of Stirling, Stirling FK9 4LA, UK

**Keywords:** cyanobacteria, co-occurrence, toxicity, plastics, metals, biocide

## Abstract

Toxin-producing cyanobacteria in aquatic, terrestrial, and aerial environments can occur alongside a wide range of additional health hazards including biological agents and synthetic materials. Cases of intoxications involving cyanobacteria and cyanotoxins, with exposure to additional hazards, are discussed. Examples of the co-occurrence of cyanobacteria in such combinations are reviewed, including cyanobacteria and cyanotoxins plus algal toxins, microbial pathogens and fecal indicator bacteria, metals, pesticides, and microplastics. Toxicity assessments of cyanobacteria, cyanotoxins, and these additional agents, where investigated in bioassays and in defined combinations, are discussed and further research needs are identified.

## 1. Introduction

Research on the production, properties, monitoring, and analysis of toxigenic cyanobacteria, and of particular cyanotoxins, has increased greatly over the past 40 years [1,2,3,4,5]. Aspects of the environmental occurrence, biosynthesis, properties, and health significance of the most widely investigated individual classes of cyanotoxins, principally the microcystins, nodularins, saxitoxins, cylindrospermopsins, and anatoxins have been reviewed [6,7,8,9,10,11,12]. On the toxicity assessment of cyanobacterial cultures and environmental samples containing cyanobacteria, and the involvement of specific cyanotoxins, the research has passed through several discernible phases. The earliest known experimental investigations into the suspected toxicity of cyanobacteria, which followed animal poisonings, were performed by George Francis [13,14]. These involved the oral dosing of healthy animals, of the same species which had succumbed to intoxication, with the suspected toxic material, in this case *Nodularia* scum, and the consequent replication in the dosed animals of mortalities and gross signs of organ damage. Bioassays, a quantitative development of this logical approach, but initially without the chemical identification and quantification of the toxic substances in the test material, were then increasingly used to test for toxic principles for several decades [1,2,3]. These were gradually complemented, and then overtaken by the emerging methods for the identification and quantification of specific cyanotoxins in the test material and the quantitative characterization of toxicity using individual purified cyanotoxins originally obtained via bioassay-guided purification of cyanobacterial material. The increasing availability of physico-chemical methods for analysis and cyanotoxin purification, especially for microcystins, nodularin-R, saxitoxins and anatoxin-a, and later for cylindrospermopsins [1,2,3,4,5], appears to have led to an increased focus on the use of these methods, with a corresponding decline in the use of the whole animal bioassays. The move away from whole animal bioassays has been further influenced by increasing ethical and humanitarian concerns. Although non-mammalian in vivo and biochemical, enzyme-based, and cell-based in vitro bioassays are available [3,15] the continuing focus on the use of physico-chemical methods has also been influenced by the increasing need for validated, quantitative procedures to satisfy statutory regulations and guidelines [16]. In addition, genetic methods [17] may also contribute to a reduction in bioassay use through providing alternative testing to understand the potential for toxin production by cyanobacteria.

The further development and application of physico-chemical analytical methods for the detection, identification, and quantification of specific cyanotoxins continues to support and enable the application of policies for the risk management of water resources affected by cyanobacterial mass populations. These include specific methods for individual classes of cyanotoxins [5] and, increasingly, methods for the multiclass analysis of the toxins in single procedures [18,19,20,21]. However, whilst such methods alone can provide a partial indication of toxicity presented by axenic strains of cyanobacteria grown in the laboratory, they may not take into full account the toxicological significance of mass populations of cyanobacteria in open environments.

**Figure 1:**
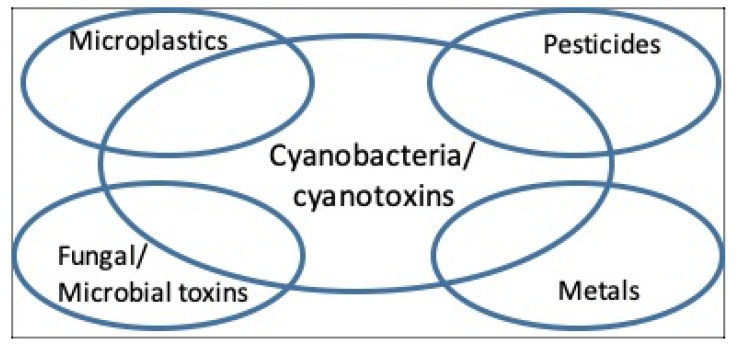
Examples of interactions of cyanobacteria with other potentially toxic components of water.

In marine and freshwater environments and in terrestrial and aerial habitats, whilst cyanobacteria can readily appear as “dominant”, i.e., to account for the majority of the microbial biomass, they can be accompanied by a wide range of other microbes including microalgae, chemoheterotrophic bacteria, and protozoa [22,23]. Where such environments are subjected to intensive anthropogenic human pressures (e.g., wastewater and human sewage disposal and industrial discharges), the dominant cyanobacteria can co-occur with additional biological toxins (e.g., [19]), chemical pollutants e.g., metals [23], and with pathogenic microbes [24]. Examples of the co-occurrence of cyanotoxins, plus other chemical agents and microbial pathogens, and cases of co-exposure are reviewed (Figure 1). Research to further understand the health significance of cyanotoxins is discussed in a broader context of cyanotoxin co-occurrence and co-exposures with additional biological and anthropogenic toxic agents.

It is well established that when exposure to toxic compounds involves mixtures of two or more toxic substances, the effect of such mixtures on susceptible individuals or populations can be unpredictable, but different from the exposures to each of the toxins if applied separately. Examples of the three possible outcomes, namely additive, synergistic, and antagonistic effects [25] are increasingly emerging, where bioassays including toxigenic cyanobacteria, and specifically when purified cyanotoxins and non-cyanobacterial toxic agents, are performed (Table 1).

Of the classes of toxins produced by cyanobacteria, with the exception of guanitoxin (formerly anatoxin-a(*S*) [41]), all encompass multiple chemical structures, ranging from a few (e.g., anatoxin-a and homoanatoxin-a) to hundreds, in the case of microcystins (>240, [42]). Similarly, analyses of blooms and cultures of cyanobacteria have shown that multiple variants can exist in extracts of individual strains, such as for *Microcystis* PCC7820 with at least 10 microcystin variants reported [43] and a Thai strain of *Cylindrospermopsis* that has been shown to produce two additional variants of cylindrospermopsin [44]. Therefore, when toxicity assessment is performed, then although classes of cyanotoxins may be identified, the differing amounts of variants, whether of e.g., microcystins or anatoxins, each with potentially differing toxicities when determined individually (e.g., [45]), may influence the risk assessment of such bloom material. On occasion, some strains have been reported to include multiple classes of toxins, such as microcystins and guanitoxin, as in *Anabaena* 525-17 [46]. In addition to these cyanotoxins, all cyanobacteria characteristically produce lipopolysaccharide endotoxins [47].

Fitzgeorge et al. [26] provided early evidence of the synergistic action of two purified cyanotoxins (anatoxin-a and microcystin-LR) when administered intranasally to mice. In this case, the administration of a sublethal dose of microcystin-LR 30 min before that of anatoxin-a lowered the LD_50_ of the latter four-fold. Perhaps the most high-profile human poisoning event occurred at Caruaru, Brazil [48,49]. Extensive investigations into the deaths of 50 people at a hemodialysis clinic identified microcystins as the most likely principal cause of the fatalities, although several symptoms were reported by those affected. Investigations continued into the intoxications and cylindrospermopsin was also implicated, suggesting the possibility of multiple cyanotoxin exposures [50].

Clearly, when bioassays are performed in vivo and in vitro with an individual purified cyanotoxin or defined combinations of purified cyanotoxins, then the responses can be unequivocally ascribed to the toxin(s) administered. However, since cyanobacterial cells, whether in axenic monocyanobacterial culture or in environmental samples, can produce a range of cyanotoxins and other bioactive secondary products, then bioassays with crude extracts from such sources unavoidably involve exposures to mixtures of toxic agents. These may be exclusively of cyanobacterial origin when derived from monocyanobacterial axenic cultures, or from cyanobacterial plus a wide range of toxins/toxicants from additional biological and anthropogenic sources in the case of environmental samples.

## 2. Environmental Intoxications Involving Toxigenic Cyanobacteria and Additional Agents

Some indications are available of contributory exposure to environmental health hazards in addition to toxigenic cyanobacteria or specific cyanotoxins in wildlife poisoning episodes. Thus, whilst a major role for microcystins and anatoxin-a in the mass mortalities of Lesser Flamingos (*Phoeniconaias minor*) at Kenyan lakes was identified, additional contributions due to heavy metals, pesticides, and mycobacterial infection were likely to have occurred [51,52]. A major role of microcystins was similarly deduced from cyanotoxin analyses and pathology in the deaths of Mute Swans (*Cygnus olor*) in the UK, although the additional contribution of lead was inferred from the abundance of fishermen’s lead sinker-pellets in the birds’ stomachs [53]. Microcystin-, saxitoxin-, and cylindrospermopsin-producing cyanobacterial blooms were indicated to have been early contributors to an extended major fish-kill in the Lower St. John’s River, Florida. Later factors arising from cyanobacterial decomposition and lysis included lower dissolved oxygen concentrations, elevated ammonia concentrations, and hemolytic contribution from the dinoflagellate alga *Heterosigma akashiwo* [54].

Cases of human illness associated with exposure to toxigenic cyanobacteria also indicate the possible contribution of additional factors. In 1979, a severe hepato-enteritis outbreak occurred among consumers (mainly children) of drinking water from a Palm Island reservoir in Queensland, Australia, containing a bloom of *Cylindrospermopsis raciborskii* [55]. The subsequent isolation and characterization of cylindrospermopsin from *C. raciborskii* from the reservoir provided strong evidence for a contributing role of the cyanotoxin in the Palm Island illnesses. However, as pointed out by Hawkins et al. the evidence suggested that the cylindrospermopsin-producing bloom should be considered as only one possible cause [55]. It is unknown whether the copper concentration in the water, arising from the prior treatment to kill the *C. raciborskii* bloom, also contributed to the illness. An episode requiring hospitalization of army cadets undergoing swimming and canoeing exercises at Rudyard Lake, northern England, occurred after the cadets ingested microcystin-containing *Microcystis aeruginosa* scum [56]. Atypical pneumonia, liver damage, and blistering around the mouth were attributed to microcystin ingestion. However, whilst no evidence for enterovirus contamination of the water was apparent, *Escherichia coli* counts indicated that the water was unsuitable for bathing [56].

## 3. Additional Health Hazards: Their Co-Occurrence and Interactions with Cyanobacteria and Cyanotoxins

A wide range of microbial and chemical health hazards exists with which cyanobacteria can be associated. The associations range from the use of purified materials in experimental laboratory designs to investigate combined toxicological responses in vitro and in vivo, to the co-occurrence of multiple hazards in open environments.

### 3.1. Fungal and Algal Toxins

The health significance of human exposure to the fungal toxin aflatoxin B1 (via the diet), plus to microcystin via drinking water, was recognized almost 30 years ago in China [57,58,59]. A high incidence of primary liver cancer among local populations using surface drinking water containing microcystin-producing cyanobacteria was associated with additional chronic exposure to the hepatocellular carcinoma risk factor aflatoxin B1 via food consumption, including moldy maize. Further risk factors were presented by Hepatitis B virus and alcohol [60]. Whilst not characterized as a primary carcinogen itself, the tumor-promoting action of the microcystin(s) was expressed by the chronic exposure of the population to the cyanotoxin, and to the primary carcinogenicity of aflatoxin B1 and/or the Hepatitis B virus. These milestone discoveries in the history of cyanotoxins research and risk management [6,8,9,57,58,59], not only illustrate the importance of exposure to cyanotoxins plus additional health hazards, but also provide an example of the hazards of such combined exposure via multiple exposure media: in this case, the media being water and food. Whether the timing of the exposure to microcystin-containing cyanobacterial blooms and to aflatoxin-B1, i.e., as co-exposure or sequential exposure, influenced the outcome among the human population, remains less certain. Trials using human hepatic cells in vitro and rats have indicated that whilst the tumor promoting actions of microcystin were confirmed, antagonistic action can occur when the cyanotoxin at low dose is co-applied together with the fungal toxin [33].

The co-occurrence of cyanotoxins with microalgal phycotoxins has received little attention. Blooms and shoreline scums of several species of toxigenic marine microalgae have long been recognized for their roles in the mass mortalities of fish, seabirds, and sea mammals and in human intoxications, from mild to fatal, via shellfish consumption [2]. For reviews concerning marine microalgal phycotoxins, including the saxitoxins, palytoxins, ciguatoxins, brevetoxins, domoic acid, okadaic acid, pinnatoxins, yessotoxins, and azaspiracids, see [61,62,63]. No evidence for the production of most classes of cyanotoxins by microalgae is available, namely of microcystins, nodularins, cylindrospermopsins, anatoxin-a, or guanitoxin. In addition, the lipopolysaccharide (LPS) endotoxins, being structural components of Gram-negative prokaryotes are characteristic of cyanobacteria [47] but are apparently lacking in eukaryotic microalgae. Thus, for these classes of cyanotoxins to co-occur with the potent phycotoxins of marine waters, it is necessary (i) for toxigenic cyanobacteria to also be present and growing in the environment, or (ii) for the cyanotoxins to be introduced into the microalgal environment. Indeed, such an introduction occurred in the Monterey Bay National Marine Sanctuary, California, where microcystin-producing *Microcystis* blooms entered the Bay waters from inland freshwaters via river inflows. Mass deaths of sea otters (*Enhydra lutris nereis*) occurred due to the consumption of marine shellfish which had accumulated the microcystins from the river inflows [64]. In this case, analyses for other candidate cyanotoxins (nodularin and anatoxin-a) and for marine phycotoxins (okadaic acid and yessotoxin) were negative. Another area of California, namely San Fransisco Bay, has shown the presence of multiple toxin classes, often detected in molluscs [65]. From an analysis of mussels, microcystins, domoic acid, diarrhetic shellfish toxins, and paralytic shellfish toxins were identified with all four toxin classes detected in 37% of mussels.

However, some classes of cyanotoxins are not exclusively the products of cyanobacteria [66]. Saxitoxins have long been known to be produced by marine dinoflagellates, including species of *Alexandrium*, *Gymnodinium*, and *Pyrodinium* [63] and by several strains of cyanobacterial species, including *Aphanizomenon* spp., *Dolichospermum circinale* (formerly *Anabaena circinalis*), *Cylindrospermopsis raciborskii*, *Raphidiopsis brookii*, *Lyngbya wollei* [67], and *Scytonema crispum* [68,69]. The neurotoxic diaminoacids, β-*N-*methylamino-l-alanine (BMAA), *N-*(2-aminoethyl)glycine (AEG), and 2,4-diaminobutyric acid (2,4-DAB), with LC-MS/MS used to confirm analytical specificity, also appear to have multiple origins, including diverse cyanobacteria, marine and freshwater diatoms, and a brackish coastal water dinoflagellate [70,71,72,73,74,75,76,77], and these toxins can also be present along with microcystins and brevetoxins [78]. Wider origins of these neurotoxins are also indicated by the presence of BMAA in the peptides found in chemoheterotrophic bacteria including environmentally widespread *Paenibacillus* spp. [79,80].

### 3.2. Microbial Pathogens

A close association of cyanobacteria, including toxigenic species, typically occurs with other microbes in aquatic and terrestrial environments [22,23]. The close proximity of the cyanobacteria and their non-phototrophic, mutualistic partners can enable the two-way exchange of metabolites and nutrients and provide a protective physical substrate for bacterial attachment and gene transfer. Cyanobacterial extracellular polysaccharides and glycoprotein sheath materials can provide a substrate for the bacterial attachment [23]. In addition to the association with a wide range of non-pathogenic bacteria (e.g., [81]), marine, estuarine, and freshwater cyanobacteria can form close associations with human pathogenic bacteria. When large blooms of cyanobacteria occur and people are exposed to such blooms, then health complaints and non-specific symptoms are often reported [82]. Such symptoms can include fever, pneumonia, and headaches as examples (reviewed in [82]). Therefore, exposure to cyanobacterial blooms may have the added issue of multiple insults affecting human health, especially in those individuals with underlying medical conditions.

In 1884, Robert Koch, the discoverer of the cholera bacillus, suggested from field studies that “aquatic flora” might serve as environmental reservoirs of cholera [83]. Indeed, searches for possible reservoirs of survival of toxigenic *Vibrio cholerae* 01 in a pond in Dhaka, Bangladesh, used for bathing, swimming, washing, and drinking, indicated the seasonal survival of the pathogen in the extracellular mucilage of the cyanobacterium *Anabaena variabilis* [84]. Whilst the specificity of this association is not extended to euglenoid microalgae, it does extend to the mucilaginous masses of other cyanobacteria, including *Microcystis* colonies [85] and microcystin-producing *Oscillatoria* filaments [86]. Without the application of more effective cyanobacterial risk management measures, the role of cyanobacteria in providing inter-epidemic reservoirs of *V. cholerae* is likely to increase as compounding anthropogenic pressures and climate change continue to result in increases in cyanobacterial population size, seasonal duration, and geographical spread [4]. Populations of other potentially pathogenic *Vibrio* spp. have increased from 2000 to 2018 in the Neuse River Estuary, North Carolina, USA but no correlations were apparent with changes in temperature, salinity, or dissolved oxygen concentration, factors which have influenced *Vibrio* abundance elsewhere [87]. The eutrophic estuary waters contain abundant blooms including microalgae and cyanobacteria [88] although whether the cyanobacteria are physically associated with the potentially pathogenic *Vibrio* spp. in the Neuse water is not apparent.

Although the waterborne human pathogenic protozoon *Cyclospora* was initially thought to be a cyanobacterium, this is not so, and the protzoon is able to cause diarrhea, sickness, and abdominal pain, similar to the protozoa *Cryptosporidium parvum* and *Giardia lamblia* [89]. An increased abundance of potential microcystin-producing cyanobacteria (*Aphanocapsa* and *Microcystis* spp.) was observed with *Cryptosporidium* and *Giardia* spp. in a reservoir supplying drinking water to the metropolitan Belo Horizonte area, southeastern Brazil [90]. It is notable that waterbody conditions which favor the persistence of *Cryptosporidium* and *Giardia* spp., including nutrient enrichment and high retention times, can also enhance cyanobacterial growth [23], increasing the potential for co-occurrence and co-exposure to pathogenic protozoa and cyanotoxins.

### 3.3. Metals

Relations between cyanobacteria and metals constitute a large field. Of toxicological relevance are (i) the chronic and acute effects of metals on the growth, metabolism, and survival of cyanobacteria and (ii) the ability of cyanobacteria to accumulate, detoxify, metabolize, and sequester metals [91,92,93]. Depending on growth conditions, the effects of dissolved metal ions (e.g., Ca, Cu, Pb, Cd) can include both the inhibition and stimulation of *Microcystis* blooms [94], and the presence of copper may affect the detoxication of e.g., nodularin by disrupting microbes that may degrade this cyanotoxin [95]. Copper is often used as a biocide to lyse cyanobacterial cells during blooms. Although successful, in the case of e.g., *Microcystis*, this has had the effect of releasing cyanotoxins from an intracellular pool to an extracellular pool with the result that liver damage may result after drinking water from which cyanobacterial cells and cell debris have been removed, but without the assured removal of extracellular microcystin [96]. Furthermore, if the copper used for lysis is not effectively removed during drinking water treatment, then this may subsequently also pose an additional toxicological burden on people and animals.

Research on metals and cyanotoxins has largely focused on the effects of iron on microcystin production. Pioneering studies on iron limitation in axenic *Microcystis aeruginosa* cultures revealed increases in microcystin-RR and -LR (MC-RR, MC-LR) production [97]. Early sampling and analytical procedures in the latter study are likely to have resulted in the combined analysis of the former intra- and extracellular pools. Further investigations into microcystin production by *M. aeruginosa*, *Microcystis novacekii*, and *Phormidium autumnale* cultures have included increases upon iron addition but also increases upon iron limitation during culture. Positive effects of copper, zinc, and manganese ions on microcystin production under conditions of metal enrichment of, and metal limitation of, cyanobacterial cultures have also been observed, possibly indicating physiological and biochemical roles of the metals in microcystin biosynthesis, in addition to a siderophore function for microcystins in metal acquisition [98]. No specific differences in anatoxin-a production by *P. autumnale* were found in response to growth under an environmentally encountered, low-to-high range of iron or copper concentrations [99].

Direct interaction between cyanotoxin molecules and metals has been investigated in vitro with two classes of cyanotoxins. The purified post-synaptic neuromuscular blocking neurotoxin, BMAA, is a potent chelator of divalent metal cations including copper and zinc [100]. Whether such chelation occurs in BMAA-producing cyanobacterial cells and, if so, if toxicity is thereby influenced, is not known. Divalent copper and zinc binding occur to at least three purified microcystins (MC-LR, MC-LW, MC-LF) with formation constants (K_i_) indicating that all three cyanotoxins are medium-strength metal ligands. Single amino acid substitution in the heptapeptide microcystin ring (arginine versus tryptophan, or versus phenylalanine) did not influence the strength of the metal-microcystin association [101]. Whether metal-microcystin binding influences toxicity and whether this binding occurs in the cyanobacterial producer-cells and/or in the surrounding water after microcystin release are also unknown. However, this possibility is viewed alongside the increasing evidence for the combined toxicity of microcystins and metals: co-exposure bioassays involving MC-LR and copper, at environmentally encountered concentrations, have revealed synergistic toxicity against the early development of the zebrafish, *Danio rerio*. Uptake of the toxins by the fish involved microcystin and copper transporters [35]. Toxicity assessment of BMAA and methylmercury to primary neurocortical cells show a synergistic toxicity [102] and assessment of other cyanotoxins with such organic forms of metals is required.

### 3.4. Pesticides

One cyanotoxin, the phosphorylated cyclic *N*-hydroxyguanidine, guanitoxin (anatoxin-a(S)) exerts toxicity via the irreversible inhibition of acetylcholinesterases in common with organophosphorus pesticides [6,103]. No other cyanotoxins are known to have the same modes of action as those of synthetic pesticides. The co-occurrence of cyanobacteria, and of pesticides, including herbicides, fungicides, and insecticides, is a concern in water resources with a high human usage and dependency. For example, in paddy fields nitrogen-fixing cyanobacteria can serve as a valuable biofertilizer and contribute to rice production and an aim is to reduce the negative impacts of the pesticides on cyanobacterial growth [104]. Nevertheless, the inadvertent entry of pesticides into waterbodies from human activities, especially agriculture, appears to be a ubiquitous process and numerous investigations into the inhibitory impacts of pesticides on aquatic biota have occurred. A wide-ranging survey of investigations into the effects of pesticides in aquatic microbes included the effects of insecticides, herbicides, and fungicides on the viability of cyanobacteria, with overall dose-dependent growth inhibition occurring [105]. Most of the named cyanobacteria in this survey were members of cyanotoxin-producing taxa. However, investigations to date do not appear to have included relations between pesticides and the production and impacts of cyanotoxins. In addition to the possible contribution of pesticides (and metals) alongside cyanotoxins to the mass mortalities of Lesser Flamingos in East African lakes [51,52,106], both pesticides and cyanotoxins may contribute to the marked decline in the American Alligator (*Alligator mississippiensis*) population in eutrophic Lake Apopka, Florida, with high egg failure and anomalous endocrine function [107].

The adult invertebrate grazer *Daphnia pulicaria* was exposed to the purified pesticide carbaryl (1-naphthyl methylcarbamate) plus whole cells of microcystin-producing *M. aeruginosa* at a range of sublethal concentrations [108]. The actual dose of microcystin(s) assimilated per animal was estimated from the analysis of whole *D. pulicaria* by ELISA, although the immunoassay used does not distinguish between authentic microcystin(s) and a range of microcystin detoxification products [109]. However, at a range of sublethal carbaryl concentrations, adverse effects on egg numbers per female, delayed maturation, offspring mortality, and body malformations occurred with outcomes increased by the addition of *M. aeruginosa* cells. Additive and synergistic actions between carbaryl and the microcystin-containing cyanobacterial cells were indicated [108]. Hinojosa et al. [39] have recently provided a significant example of the needed, quantitative, baseline studies on the toxicology of pesticides in co-occurrence with cyanotoxins. In vitro bioassays using the human neuroblastoma cell line SH-SY5Y evaluated the effects of individual, versus combined exposure to purified cylindrospermopsin and chlorpyrifos [*O,O*-diethyl *O*-(3,5,6-trichloro-2-pyridinol) phosphorothionate]. Cytotoxicity and mechanistic endpoint comparisons after 24 and 48 h of exposure, at environmentally relevant concentrations, indicated antagonistic action between the pesticide and cyanotoxin [39].

The herbicide glyphosate has been used widely to control plants and it has been shown to occur in waterbodies, with toxicity demonstrated [110]. Its presence in waterways is well known and has been shown to have an adverse effect on the growth of cyanobacteria, including *Microcystis* [111]. Further effects of glyphosate on cyanobacteria include enhanced extracellular release of microcystins [112] with the potential for organisms to respond to combinations of microcystins and glyphosate, such as the mussel *Unio pictorium* [113].

### 3.5. Microplastic and Nanoplastic Particles and Contaminants

The global occurrence of microscopic plastic particles (microplastics) throughout the world’s oceans has been recognized for several years [114] and their further occurrence in human feces, freshwater lakes and rivers, groundwater, and estuaries is becoming increasingly recognized [115,116]. Cyanobacteria in both marine and freshwaters are among the wide range of microbes which, with mineral particles, attach to microplastics, forming a biofilm. The adhesion of microbes, with subsequent development of an extracellular polysaccharide layer, may contribute to the sinking or buoyancy properties of these complexes, and to the further sorption of metals, particularly, iron and manganese [117]. Among toxicological hazards presented by the microplastics accumulating in water resources are the endocrine-disrupting bisphenol contaminants, including bisphenols A, S, F, and AF [118]. Cylindrospermopsin is a strong candidate for toxicity evaluation in combination with bisphenols since (i) its wide geographical occurrence in freshwaters is becoming more apparent; (ii) a high percentage of the total cylindrospermopsin pool is extracellular when the *C. raciborskii* producer cells are still intact, and (iii) the cyanotoxin exhibits a wide range of actions, including hepatotoxicity, inhibition of protein synthesis, genotoxicity, and potential carcinogenicity [6,9,40]. A complex of interactions between purified cylindrospermopsin and bisphenols was found in in vitro bioassays using HepG2 cells. Whilst bisphenols alone reduced cell viability or induced DNA double strand breaks, antagonistic activity of bisphenol against these actions by cylindrospermopsin was indicated. However, further possible additive or synergistic effects on HepG2 gene deregulation were also indicated by co-exposure to the bisphenols plus cylindrospermopsin [40]. No bioassays involving purified microcystins with micro- or nanoplastics are yet apparent, although toxicologically significant interactions have been reported between nanoplastics and *Microcystis aeruginosa* cells [118], and laboratory-ware plastics are also known to bind microcystins from solution [119,120] and plastic nanoparticles may also bind glyphosate [111]. Amino-modified polystyrene nanoplastics increase microcystin production by the cyanobacterial cells and also increase the extracellular release of the toxin(s) according to microcystin immunoassay. The prevalence and proximity of microplastics, nanoplastics, and cyanobacteria [115,116,117], and widespread ability of the latter to produce microcystins [5,8,9,11,12] require further research into the toxicological interactions of these ubiquitous synthetic and naturally occurring, biological health hazards.

## 4. Implications for Water Safety Guidelines, Legislation, and Water Treatment

The need to provide safe recreational and bathing waters and drinking water provides multiple challenges due to the potential for combinations of toxic compounds, with possible synergistic and additive effects, to occur at concentrations deemed unsafe for human health [121,122]. In order to prevent potential adverse toxicities arising from exposure to multiple toxins, to cyanotoxins plus microbial pathogens, and to cyanotoxins plus more recently recognized health hazards including microplastics, the ability of water safety guideline values (GV) and legislation to provide adequate safety margins in the event of potential multiple exposures need to be verified. In several cases, the same drinking water treatment technologies are used for the removal and/or destruction of cyanotoxins, and of other toxicants (Table 2).

Consequently, in order to prevent or reduce toxicity during events when co-occurrence arises, then the efficacy of such multipurpose water treatment technologies needs to be verified, and increased where needed, with respect to each of the classes of cyanotoxins and of additional toxicants in the raw water. Furthermore, the development and implementation of advanced water treatment technologies should take into account the potential for real-world exposures to a wide range of toxicants and scenarios, from e.g., natural occurrence [121], to deliberate or man-made additions [136]. Such events can potentially occur alone or in combination and during times of high-water usage which may include cyanobacterial blooms in source waters, the potential for multiple exposures should be acknowledged and contingency plans should consider this issue. Furthermore, often contingencies may include the switching of source waters and if different, but potentially hazardous situations exist in these waters, then alternative or enhanced treatment technologies, or e.g., the temporary provision of bottled waters, may be required.

Understanding of the health risks presented by cyanotoxins has progressed considerably [6,7,8,9], but the risk management of microcystins, and of microcystin-producing cyanobacteria has been the focus of guideline derivation for health protection [137]. GVs in addition to those for microcystins, including for cylindrospermopsin [121,134] are also being derived. Although GVs for microplastics do not exist, the WHO has derived GVs for six monomers which can leach from the plastics, ranging from 0.3 to 300 μg/L [138]. Some metals and pesticides also have GVs for drinking water and are also generally in the low μg/L range [139]. Whether the GV values, with in-built safety margins, accommodate the additive and synergistic toxicities which can arise due to cyanotoxin co-occurrences and to the co-occurrence of cyanotoxins with other toxicants including microplastics derivatives (Section 3.5) merits investigation.

## 5. Concluding Remarks

Following wildlife-, domestic animal-, and human-intoxications due to exposure to cyanobacterial mass populations, the volume of research over recent years into the toxicology and toxinology of cyanobacteria and cyanotoxins has increased greatly and such growth continues e.g., [1,5,7,8]. In recognition of the co-occurrence of multiple variants within individual classes of cyanotoxins, of different cyanotoxin classes, and of cyanotoxins plus phycotoxins, it is encouraging that physico-chemical methods for the co-analysis of these combinations are being developed [5,19,20,21,140]. However, since cyanobacterial mass populations commonly develop in waterbodies which are under intensive anthropogenic use (e.g., for domestic, industrial, and agricultural wastewater discharge, abstraction for drinking water treatment, recreation, crop irrigation, and fisheries) it should be anticipated that toxigenic cyanobacteria can co-occur with a wide range of additional biological and chemical health hazards. Indeed, sufficient examples exist, reviewed here, of animal and human health incidents and intoxications associated with multiple health hazards including cyanobacteria and cyanotoxins, and other contributory biological toxins, microbial pathogens and anthropogenic chemical products. Whilst further research on the toxicity of the established and emerging cyanotoxins via bioassays is needed, including defined cyanotoxin combinations and environmental materials [15], more cyanotoxin bioassays including other environmental toxins and toxicants, as exemplified in Table 1, are required. Data on the toxicity of cyanotoxins in combination with other biotoxins and chemical toxic compounds, may then contribute to the assessment of whether guideline values and legislation for health protection can also accommodate multiple exposure to cyanotoxins plus the other biological and chemical agents. The route of administration of cyanotoxins may also affect the toxicological outcome or the mixtures of compounds that may be present, such as lead and particular matter (PM_2.5_ and PM_10_) that can occur as airborne components [141,142], potentially in addition to cyanotoxins [143].

The recognition of the multiple occurrence and combined toxicity of cyanotoxins, plus additional toxicants and pathogens, is a growing area of research and such co-occurrences may increase further with the growth of the human population, increasing demands upon water resources and climate change. However, such co-occurrence(s) may have already adversely affected human health in an earlier period. High concentrations of mercury, of phosphate consistent with eutrophic conditions, plus 16S rRNA amplicons indicating *Planktothrix* and *Microcystis* blooms (and thus potentially microcystins), have been found in dated sediment profiles from former reservoirs serving the ancient former Mayan city of Tikal in Guatemala. This potentially toxic combination may have contributed to the demise of the Mayan population and of the city in the ninth Century CE [144].

## Figures and Tables

**Table 1:** Examples of toxicity assessment by bioassay of purified cyanotoxins in combinations and of cyanotoxins plus additional environmental toxins/pollutants.

Toxic Agents	Test Organism/Cell Line	Outcome ^1^	Ref.
MC-LR plus Antx-a	Mouse, intranasal bioassay	SYN	[26]
MC-LR plus Antx-a	*Selenastrum capricornutum*	SYN	[27]
MC-LR plus Antx-a	*Cyprinus carpio*, carp cells	SYN (potential)	[28]
MC-LR plus Antx-a	*Vallisneria natans*, and microbial biofilm	ANTAG	[29]
MC-LR plus *Microcystis* LPS	*Artemia salina* and *Daphnia* sp.	ANTAG	[30]
MC-LR plus CYN	*Chlorella vulgaris*	SYN	[31]
MC-LR plus cyanobacterial λ-linolenic acid	*Daphnia magna*	ADDIT	[32]
MC-LR plus aflatoxin B1	Human hepatic cells	ANTAG	[33]
MC-LR plus aflatoxin B1, and plus fumonisin B1	HepG2, Caco2, MDBK cell lines	ADDIT, SYN, ANTAG	[34]
MC-LR and copper	*Danio rerio*	SYN	[35]
MC-LR plus copper	*Vallisneria natans*	SYN	[36]
MC-LR plus linear alkyl- benzene sulphonate	*Lactuca sativa*	SYN	[37]
MC-LR plus phenanthrene	*Lemna gibba*	ANTAG, SYN, ADDIT	[38]
CYN plus chloropyrifos	Human SH-SY5Y neuroblastoma cell line	ANTAG	[39]
CYN plus bisphenols	HepG2 cells	SYN, ADDIT	[40]

^1^ SYN, synergistic; ADDIT, additive; ANTAG, antagonistic toxicological outcomes of applications of multiple toxic agents. MC-LR, microcystin-LR, Antx-a, anatoxin-a; CYN, cylindrospermopsin. Outcomes in several systems were influenced by relative concentrations, and in some cases by timing of applications of toxins/toxicants, see references.

**Table 2:** Examples of water treatment processes for the removal of potentially toxic compounds.

Toxicant	Treatment Process	References
Fungal/microbial toxins	SF, C	[123]
Pesticides	Oz, AC,	[124,125,126]
Microplastics	C, U	[127,128]
Metals	C, A	[129,130]
Microcystins	AC, Ox, Oz, Cl	[121,131]
Anatoxin-a	Oz, Ox, Cl	[132]
Saxitoxins	AC, Cl	[133]
Cylindrospermopsin	AC, Oz, Cl	[134,135]

AC, activated carbon; Ox, oxidation; Ozonation; Cl, chlorination; C, coagulation; A, adsorption; U, ultrafiltration; SF, sand filtration.
